# Minimum Lateral Bone Coverage Required for Securing Fixation of Cementless Acetabular Components in Hip Dysplasia

**DOI:** 10.1155/2017/4937151

**Published:** 2017-02-19

**Authors:** Masanori Fujii, Yasuharu Nakashima, Tetsuro Nakamura, Yoshihiro Ito, Toshihiko Hara

**Affiliations:** ^1^Department of Orthopaedic Surgery, Japan Community Health Care Organization (JCHO), Kyushu Hospital, 1-8-1 Kishinoura, Yahatanishi-ku, Kitakyushu 806-8501, Japan; ^2^Department of Orthopaedic Surgery, Graduate School of Medical Sciences, Kyushu University, 3-1-1 Maidashi, Higashi-ku, Fukuoka 812-8582, Japan

## Abstract

*Objectives.* To determine the minimum lateral bone coverage required for securing stable fixation of the porous-coated acetabular components (cups) in hip dysplasia.* Methods.* In total, 215 primary total hip arthroplasties in 199 patients were reviewed. The average follow-up period was 49 months (range: 24–77 months). The lateral bone coverage of the cups was assessed by determining the cup center-edge (cup-CE) angle and the bone coverage index (BCI) from anteroposterior pelvic radiographs. Further, cup fixation was determined using the modified DeLee and Charnley classification system.* Results.* All cups were judged to show stable fixation by bone ingrowth. The cup-CE angle was less than 0° in 7 hips (3.3%) and the minimum cup-CE angle was −9.2° (BCI: 48.8%). Thin radiolucent lines were observed in 5 hips (2.3%), which were not associated with decreased lateral bone coverage. Loosening, osteolysis, dislocation, or revision was not observed in any of the cases during the follow-up period.* Conclusion.* A cup-CE angle greater than −10° (BCI > 50%) was acceptable for stable bony fixation of the cup. Considering possible errors in manual implantation, we recommend that the cup position be planned such that the cup-CE angle is greater than 0° (BCI > 60%).

## 1. Introduction

Developmental dysplasia of the hip (DDH) is a common cause of hip osteoarthritis [1], characterized by insufficient acetabular coverage on the femoral head and shallow acetabular concavity [2]. During cementless total hip arthroplasty (THA) in patients with DDH, a hypoplastic acetabulum often makes it difficult to obtain sufficient bone coverage and initial stability of the acetabular component (cup), especially in cases of severe dysplasia [3].

Several techniques have been reported to manage insufficient bone coverage, including structural autograft [4], superior placement of the cup [5], and medialization of the cup [6]. However, the minimum bone coverage required on the porous-coated cup for securing stable fixation without these special techniques remains unclear, since the previously reported values vary greatly among studies, ranging from 50% to 80% [7–14], and adequate evidence is not available to determine the absolute value.

Thus, the purpose of the present study was to determine the effect of lateral bone uncoverage on the fixation of the porous-coated cups and the minimum requirement for lateral bone coverage on the cup.

## 2. Patients and Methods

### 2.1. Patients

We reviewed the clinical and radiographic data of 260 consecutive patients (281 hips) with hip osteoarthritis who underwent primary THA using cementless components between April 2010 and March 2013. The institutional ethics committee of JCHO Kyushu Hospital and Kyushu University Hospital approved this study. The inclusion criterion for this study was hip osteoarthritis secondary to hip dysplasia, defined as a lateral center-edge angle of Wiberg [15] of less than 20° on anteroposterior pelvic radiographs. Of the 260 patients, 224 patients (243 hips) met this criterion. The exclusion criteria included lack of a minimum 24-month follow-up, prior acetabular osteotomy, and other hip diseases. Therefore, 4 patients (5 hips) with a history of acetabular osteotomy and 20 patients (22 hips) who had not been followed up for a minimum of 24 months were excluded from this study (follow-up rate: 91%). One patient (1 hip) with a structural bone graft was also excluded.

A total of 199 patients (215 hips) were finally eligible for this study. They included 31 men and 168 women with an average age of 66.8 ± 8.4 years (range: 50–85 years). The average body mass index was 24.9 ± 3.8 kg/m^2^ (range: 14.6–37.8 kg/m^2^). The average follow-up period was 49.4 ± 11.9 months (range: 24–77 months). According to the classification system of Crowe et al. [3], 175 hips were classified as type I, 29 hips were classified as type II, 10 hips were classified as type III, and 1 hip was classified as type IV. Fifteen patients (15 hips) had a history of femoral osteotomy. Sixteen patients had staged bilateral procedures during the study period. The patients were evaluated preoperatively, at 3, 6, and 12 months postoperatively, and annually thereafter, both radiographically and clinically. Radiographic evaluations included anteroposterior pelvic and cross-table lateral view radiographs. Further, their Merle d'Aubigné and Postel hip scores were recorded for clinical evaluation.

### 2.2. Surgical Procedures

Total hip arthroplasties were performed through the posterolateral approach in 209 hips and through the trochanteric approach in 6 hips. The cups were implanted using the line-to-line technique, and initial stability was obtained in all hips. One to 3 screws (average: 2) were used to secure the initial fixation of the cup. In 29 hips, morselized allografts from the resected femoral head were applied to fill the gap between the host bone and the uncovered portion of the cup. A concomitant subtrochanteric shortening osteotomy was performed in 3 patients (3 hips). Patients were routinely allowed full weight bearing starting on postoperative day 1. Those who underwent THAs through the trochanteric approach or who had intraoperative femoral fractures were allowed weight bearing from 4 weeks after surgery.

### 2.3. Implants

A titanium arc-splayed titanium cup with hydroxyapatite coating (AMS HA Cup; Kyocera, Osaka, Japan) [18] and a cross-linked ultra-high-molecular-weight polyethylene liner (Aeonian 910 AMS Liner; Kyocera, Osaka, Japan) were used in all hips. The bearing couples were ceramic on polyethylene in 193 hips and metal on polyethylene in 22 hips. The femoral head diameters were 26 mm in 4 hips, 28 mm in 96 hips, and 32 mm in 115 hips. Two cementless stems, namely, Perfix910 HA-coated stem (Kyocera, Osaka, Japan) and S-ROM (DePuy, Warsaw, IN, USA), were used in 193 hips and 22 hips, respectively.

### 2.4. Radiographic Evaluations

**Figure 1:**
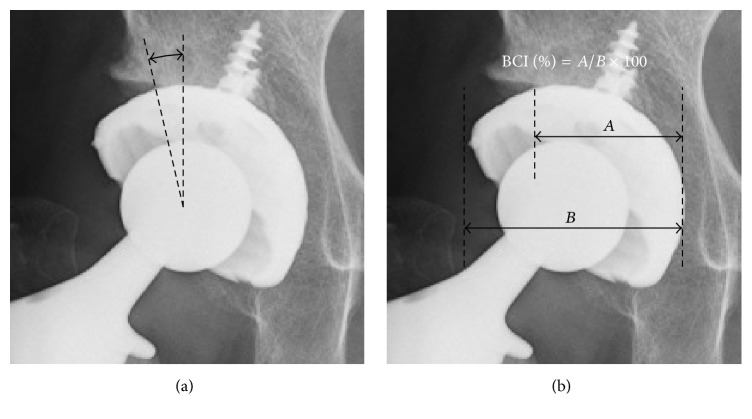
The cup center-edge (cup-CE) angle and bone coverage index (BCI) were determined on postoperative anteroposterior pelvic radiographs using the interteardrop line as a reference. (a) The cup-CE angle was determined as the angle created by the intersection of a line connecting the hip center and the lateral edge of the host bone and the line perpendicular to the interteardrop line. (b) The BCI was determined as the percentage of the horizontal width of the cup covered by the host bone (distance* A*) in the horizontal width of the cup (distance* B*).

The cup center-edge (cup-CE) angle and the bone coverage index (BCI) were measured on postoperative anteroposterior pelvic radiographs as indices of the degree of lateral bony coverage on the cups [7, 19] (Figure 1). The radiographic anteversion and inclination angles of the cups were determined with the interteardrop line as a baseline [20, 21]. The horizontal and vertical distances from the tip of the ipsilateral teardrop to the hip center were measured with the interteardrop line as a baseline. Hips with a vertical distance exceeding 35 mm were defined as having a high hip center [22].

Cup fixation was determined on the radiographs at the latest follow-up on the basis of the modified classification system of DeLee and Charnley [16, 17] (Table 1). Osteolysis was defined as a circular or oval area of distinct bone loss. Heterotopic ossification was graded according to the classification system of Brooker et al. [23]. Morselized allografts were judged as incorporated if continuity of the trabecula was found between the host bone and the graft and they were judged as absorbed if the allograft had disappeared.

Thus, the clinical results, the radiographic results of the porous-coated cup, and the association between lateral bone coverage and cup fixation were analyzed using the data described above.

### 2.5. Statistical Analysis

Student's* t*-tests, Welch's* t*-tests, or Wilcoxon rank sum tests were used to compare continuous parameters between any 2 groups, depending on data distribution (Shapiro-Wilk* W* test and* F*-test). Chi-square tests or Fisher's exact tests were used to compare categorical parameters, as appropriate. Correlation between the cup-CE angle and the BCI was evaluated using a linear regression analysis. The significance level was set at *p* < 0.05 for all tests. Statistical analyses were performed using JMP® Version 11.0 (SAS Institute Inc., Cary, NC, USA).

## 3. Results

### 3.1. Clinical Results

The average Merle d'Aubigné and Postel hip score improved from 9.8 (range: 4–16) preoperatively to 16.5 (range: 12–18) at the time of the latest follow-up. No postoperative dislocation or symptomatic thromboembolic events occurred. Further, no cases of revision for any reason were noted during the study period. Complications occurred in 8 hips: 3 hips had intraoperative femoral metaphysis fractures and concomitant wiring was performed on 2 hips. Two hips had superficial infection, which healed conservatively. Two hips had postoperative fractures of the greater trochanter after the patients fell, and these healed conservatively. Finally, 1 hip showed nonunion of the trochanteric osteotomy site.

### 3.2. Radiographic Results

At the time of the latest follow-up, all cups were determined to show stable fixation by bone ingrowth: 210 hips (97.7%) were judged as type IA and 5 (2.3%) as type IB (Table 2). Of the type IB hips, 2 showed thin radiolucent lines in zone I and 3 showed them in zone II. No cases of pelvic or femoral osteolysis or stem loosening were found. Heterotopic ossification occurred in 30 hips (14.0%), and the morselized allograft was incorporated in 27 of 29 hips (93.1%) (Table 2).

The average anteversion and inclination angles of the cup were 16.2° and 42.4°, respectively (Table 2). The average position of the hip center was 33.5 mm laterally and 21.0 mm superior to the tip of the ipsilateral teardrop, and 8 hips (3.7%) were determined to have a high hip center.

**Figure 2:**
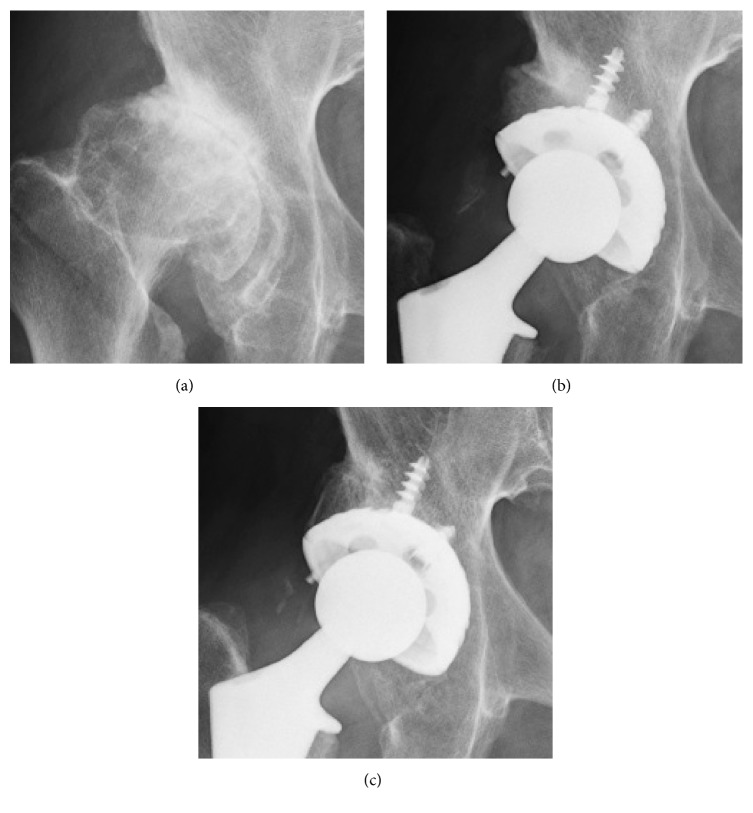
(a) Preoperative radiograph of a 54-year-old woman with Crowe type II dysplasia. (b) On a postoperative radiograph, the cup center-edge angle was −9.2° and the bone coverage index was 48.8%. A morselized autograft was used. (c) A radiograph taken 3 years after surgery showed fixation by bone ingrowth with no radiolucent lines. The allograft was perfectly incorporated into the host bone.

The average cup-CE angle and BCI were 15.8° and 75.5%, respectively (Table 2), while the minimum cup-CE angle and BCI were −9.2° and 48.8%, respectively (Figure 2). The cup-CE angle was less than 10° in 57 hips (26.5%) and less than 0° in 7 hips (3.3%). The BCI was less than 70% in 60 hips (27.9%) and less than 60% in 6 hips (2.8%). The cup-CE angle and BCI had a linear relationship (BCI = 61.8 + 0.87 × cup-CE angle, *R*^2^ = 0.80, *p* < 0.0001). According to this formula, cup-CE angles of −10°, 0°, and 10° corresponded approximately to BCIs of 50%, 60%, and 70%, respectively.

### 3.3. Association between Lateral Bone Coverage and Cup Fixation

The median cup-CE angle and BCI of 5 hips (2.3%) with radiolucent lines (type IB) did not differ from those of the 209 hips (97.2%) without radiolucent lines (type IA), with the numbers available (Table 3). No radiolucent lines were observed in hips with a cup-CE angle of <0° (7 hips) or those with a BCI of <60% (6 hips). Among the demographic parameters, patients with hips with radiolucent lines were older than those without the radiolucent line (*p* = 0.0404). Other demographic, surgical, and radiographic parameters showed no association with the presence of radiolucent lines (Table 3).

## 4. Discussion

In DDH, a hypoplastic acetabulum often compromises sufficient bone coverage and the initial stability of the cup during THA [3]. The minimum requirement for bone coverage on the porous-coated cup to ensure stable fixation remains unclear. In the present study, all 215 cups had stable fixation by bone ingrowth at an average follow-up of 4 years, and a minimum cup-CE angle of −9.2° (BCI: 48.8%) was acceptable for stable bony fixation. Thin radiolucent lines were observed for 5 hips (2.3%), and these hips were associated with older age but not with decreased lateral bone coverage.

From our study results, we assume that a cup-CE angle of approximately −10° (BCI: 50%) indicates acceptable bone coverage for stable fixation by bone ingrowth in the short term. However, we could not determine the actual threshold (cut-off value) of minimum bone coverage required to ensure stable fixation because there were no cases with failure of fixation in our study. The previously reported minimum requirements of lateral bone coverage on the cup vary greatly among studies [7–14], probably because of differences in implant selection, patients selection, and surgical techniques. The AMS HA Cup used in this study has shown excellent outcomes at a minimum follow-up of 10 years [24]. In a study of 98 THAs using the press-fit-only technique at a mean follow-up of 7.4 years, Takao et al. [7] reported that a cup-CE angle of 8.4° (BCI: 65.5%) was adequately high for press-fit cups to resist superior directed loads and achieve bone ingrowth. Another study of 81 THAs using porous-coated cups with screws at a mean follow-up of 10.6 years reported that there was no loosening when the cup surface was in contact with more than 60% of the host bone [13]. Additionally, Y.-H. Kim and J.-S. Kim [8], in a study of 116 THAs at a mean follow-up of 9.7 years, reported that 11 hips (9%) with bone coverage of less than 60% by the host bone showed aseptic loosening, while the remaining 105 hips (91%) with bone coverage exceeding 60% showed solid fixation. Lastly, Li et al. [10] observed no cup loosening in their study of 52 THAs with bone coverage between 50% and 70% at a mean follow-up of 4.8 years, and they concluded that bone coverage of 50% is acceptable. They recommended the use of morselized allografts when the host bone coverage is less than 70%. These studies indicate that when performing THA using porous-coated cup with screws and morselized autograft, the minimum bone coverage required for securing stable fixation lies between 50% and 60%.

In the present study, thin radiolucent lines were observed for 5 hips (2.3%), and these hips were associated with older age but not with decreased lateral bone coverage, based on the numbers available. The previously reported incidence of a thin radiolucent line around the porous-coated cup ranged from 1.9% to 20% [7, 10, 13]. Although none of these radiolucent lines were progressive in the short to intermediate term, further studies are needed to confirm a possible correlation between the incidence of these radiolucent lines and cup loosening in the long term.

We adopted the cup-CE angle as a key indicator of the bony cup coverage. Previous studies used various methods to estimate the bony cup coverage radiographically, including cup-CE angle, BCI, and circumferential bone coverage. Takao et al. [7] have shown that the cup-CE angle showed the highest correlation with three-dimensional bone coverage measured on computed tomography. We also determined correlation of the cup-CE angle with BCI to collectively evaluate research results of present and previous studies. On the basis of the collective results of the present and previous studies, we recommend that the cup position be planned such that a cup-CE angle greater than 0° (BCI > 60%) is achieved. Although the cup-CE angle of approximately −10° (BCI: 50%) ensured acceptable bone coverage for stable bony fixation in the short term, this value is not recommended as a target value in preoperative planning, because errors in manual implantation can result in cup positioning different from the preoperatively planned position [25] and an unintended severe lack of bony cup coverage. Additionally, only 7 hips (3.3%) had a cup-CE angle less than 0° in the present study. We intend to perform three-dimensional preoperative planning to ensure that cup placement replicates the native hip center to the best possible extent and to move the cup template superiorly to achieve a cup-CE angle greater than 0° (BCI > 60%). Sufficient bony cup coverage by the anterior and posterior acetabular wall on the axial plane should be confirmed. In cases in which a cup-CE angle greater than 0° (BCI > 50%) cannot be achieved without a high hip center, we intend to consider special techniques such as structural bone grafting.

This study has several limitations. First, the follow-up periods were relatively short. We believe the stable fixation by bone ingrowth in the short term guarantees favorable results in the long term. A previous study showed that radiolucent lines were identified within 2 years postoperatively and no new radiolucent lines were observed thereafter [7]. However, further studies are needed to determine whether fixation definitively lasts in the long term. Second is the retrospective design of this study and we did not have the option to evaluate three-dimensional bone coverage on the acetabular component. The bone coverage on the cup was evaluated on plain radiographs, which only provide two-dimensional information. A three-dimensional analysis is required to clearly determine the extent of bone coverage on the acetabular component. However, Takao et al. [7] have shown a significant correlation between the radiographic parameters of lateral bone coverage (cup-CE angle and BCI) and three-dimensional bone coverage measured on computed tomography. As radiography is the most convenient and prevalent method used in preoperative planning and patient follow-up, we believe that our results will still be useful for clinicians planning THA.

In conclusion, a cup-CE angle greater than −10° (BCI > 50%) was acceptable to achieve stable bony fixation of the cup in the short term. Considering possible errors in manual implantation and the limited number of hips with a cup-CE angle less than 0°, we recommend that the cup position be planned such that a cup-CE angle of >0° (BCI > 60%) is achieved.

## Figures and Tables

**Table 1:** Modified DeLee and Charnley classification system for acetabular fixation [16, 17].

Fixation grade		Description
Bone ingrowth, stable	IA	No radiolucent line
	IB	Radiolucent line in 1 zone
	IC	Radiolucent line in 2 zones

Fibrous fixation, stable	II	Complete radiolucent line < 2 mm in width

Fibrous fixation, unstable	III	Complete radiolucent line ≥ 2 mm in width, progressive radiolucent line in zone III, or cup migration

**Table 2:** Radiographic parameters.

Parameters	*N* = 199 patients (215 hips)
Postoperative^*∗*^	
Cup anteversion angle (°)	16.2 (6.7; 0.0–35.0)
Cup inclination angle (°)	42.4 (6.6; 22.0–60.8)
Vertical distance (mm)	21.0 (6.5; 9.0–42.3)
Horizontal distance (mm)	33.5 (4.0; 22.5–51.2)
Cup center-edge angle (°)	15.8 (9.0; −9.2 to 41.0)
Bone coverage index (%)	75.5 (8.7; 48.8–96.2)
At the latest follow-up	
DeLee and Charnley classification^†^	
Type IA	209 (97.7%)
Type IB	5 (2.3%)
Type IC	0 (0%)
Type II	0 (0%)
Type III	0 (0%)
Zone of radiolucent line^†^ (I : II : III)	2 (0.9%) : 3 (1.4%) : 0 (0%)
Osteolysis^†^	0 (0%)
Heterotopic ossification^†^ (I : II : III : IV)	23 (10.7%) : 2 (0.9%) : 5 (2.3%) : 0 (0%)
Incorporation of morselized autograft^†^	
Complete incorporation	16 (55.2%)
Partial incorporation	11 (37.9%)
Absorbed	2 (6.9%)

^*∗*^Values are presented as mean (standard deviation; range). ^†^Values are presented as number of hips (%).

**Table 3:** Comparison of demographic and radiographic parameters between hips with and without radiolucent lines.

Parameters	Radiolucent line (−) (*N* = 210 hips)	Radiolucent line (+) (*N* = 5 hips)	*p* value
Demographic			
Age^*∗*^ (years)	67.0 (50–85)	77.0 (61–79)	0.0404
Gender^†^ (M : F)	33 : 177	1 : 4	0.7952
BMI^*∗*^ (kg/m^2^)	24.0 (17.9–37.8)	20.5 (14.6–32.7)	0.1302
Crowe classification^†^ (I or II : III or IV)	199 : 11	5 : 0	1.0000
Follow-up period^*∗*^ (months)	49 (24–77)	46 (37–57)	0.7594
Surgical			
Approach^†^ (posterior : lateral)	204 : 6	5 : 0	1.0000
Stem^†^ (Perfix910 : S-ROM)	188 : 22	5 : 0	1.0000
Number of screws^†^ (1 : 2 : 3)	3 : 196 : 11	0 : 5 : 0	1.0000
Autograft^†^	27	2	0.1357
Radiographic^*∗*^			
Cup anteversion (°)	16.9 (0.0–35.0)	14.0 (6.4–16.3)	0.1349
Cup inclination (°)	42.0 (22.0–60.8)	37.1 (34.5–44.4)	0.1136
Vertical distance (mm)	19.9 (9.0–42.3)	21.4 (11.1–25.7)	0.8872
Horizontal distance (mm)	33.6 (22.5–51.2)	35.2 (26.9–38.0)	0.5316
Cup center-edge angle (°)	16.6 (−9.2 to 41.0)	9.0 (6.3–20.8)	0.1221
Bone coverage index (%)	75.7 (48.8–96.2)	68.2 (65.0–78.0)	0.1002

^*∗*^Values are presented as median (range). ^†^Values are presented as number of hips.

## Abbreviations

DDH: Developmental dysplasia of the hip
THA: Total hip arthroplasty
Cup-CE: Cup center-edge
BCI: Bone coverage index.
